# Serum long noncoding RNA HOTAIR as a novel diagnostic and prognostic biomarker in glioblastoma multiforme

**DOI:** 10.1186/s12943-018-0822-0

**Published:** 2018-03-20

**Authors:** Sze Kiat Tan, Chiara Pastori, Clara Penas, Ricardo J. Komotar, Michael E. Ivan, Claes Wahlestedt, Nagi G. Ayad

**Affiliations:** 1 Department of Neurosurgery, Sylvester Comprehensive Cancer Center, University of Miami Brain Tumor Initiative, University of Miami Miller School of Medicine, Miami, Florida 33136 USA; 2 Department of Psychiatry and Behavioral Sciences, Center for Therapeutic Innovation, Miami Project to Cure Paralysis, Sylvester Comprehensive Cancer Center, University of Miami Brain Tumor Initiative, University of Miami Miller School of Medicine, Miami, Florida 33136 USA; 3 Department of Psychiatry and Behavioral Sciences, Center for Therapeutic Innovation, Sylvester Comprehensive Cancer Center, University of Miami Brain Tumor Initiative, University of Miami Miller School of Medicine, Miami, Florida 33136 USA

**Keywords:** Glioblastoma, Long noncoding RNA, HOTAIR, Biomarker, Cancer

## Abstract

Glioblastoma multiforme (GBM) is the most common and aggressive malignant adult primary brain tumor. Despite surgical resection followed by radiotherapy and chemotherapy, the median survival rate is approximately 14 months. Although experimental therapies are in clinical trials for GBM, there is an urgent need for a peripheral GBM biomarker for measuring treatment response. As we have previously demonstrated that the long noncoding RNA HOX Transcript Antisense Intergenic RNA, or HOTAIR, is dysregulated in GBM and required for GBM cell proliferation, we hypothesized that HOTAIR expression may be utilized as a peripheral biomarker for GBM. HOTAIR expression was measured in serum from 43 GBM and 40 controls using quantitative real-time PCR (qRT-PCR). The PCR products were subsequently subcloned into pCR™4-TOPO®TA vectors for DNA sequencing. A ROC curve was also generated to examine HOTAIR’s prognostic value. The amount of HOTAIR in serum exosomes and exosome-depleted supernatant was calculated by qRT-PCR. The relative HOTAIR expression was also investigated in 15 pairs of GBM serum and tumors. We detected HOTAIR in serum from GBM patients. HOTAIR levels in serum samples from GBM patients was significantly higher than in the corresponding controls (*P* < 0.0001). The area under the ROC curve distinguishing GBM patients from controls was 0.913 (95% CI: 0.845–0.982, *P* < 0.0001), with 86.1% sensitivity and 87.5% specificity at the cut-off value of 10.8. HOTAIR expression was significantly correlated with high grade brain tumors. In addition, *Pearson* correlation analysis indicated a medium correlation of serum HOTAIR levels and the corresponding tumor HOTAIR levels (*r* = 0.734, *P* < 0.01). We confirmed via sequencing that the amplified HOTAIR from serum contained the HOTAIR sequence and maps to the known HOTAIR locus at 12q13. The serum-derived exosomes contain HOTAIR and the purified exosomes were validated by western blot and nanoparticle tracking analysis. Importantly, our results demonstrate that serum HOTAIR can be used as a novel prognostic and diagnostic biomarker for GBM.

**Electronic supplementary material:**

The online version of this article (10.1186/s12943-018-0822-0) contains supplementary material, which is available to authorized users.

## Background

Glioblastoma multiforme (GBM) is the most common and aggressive malignant adult primary brain tumor [1]. Despite aggressive surgical resection followed by radiotherapy and chemotherapy, the median survival rate is approximately 14 months [2]. As the 5-year recurrence for GBM is nearly universal, there is an urgent need to identify novel therapies for GBM [2]. To facilitate therapeutic development for GBM, peripheral biomarkers are needed to measure treatment response and identify tumor recurrence. Once a GBM patient is diagnosed and their tumor is resected, they are monitored every other month by magnetic resonance imaging (MRI). Tumor recurrence occurs usually within 14 months as judged by MRI [3]. However, distinguishing true radiographic progression from pseudoprogression is often difficult. Pseudoprogression is similar to true progression on an MRI, without the presence of a true tumor. Some estimates suggest that pseudoprogression rates are close to 20% [4]. A serum biomarker that can be used in conjunction with MRI to distinguish pseudoprogression and true progression would greatly inform clinical decisions. For instance, if the treating physician has greater confidence that a tumor has recurred, then they can suggest surgery or another chemotherapy for a resectable tumor. Additionally, with the increased use of immunotherapy, the interpretation of post-treatment MRIs continues to be confounded by immune response and edema that could otherwise be interpreted as tumor progression [3]. However, few peripheral biomarkers for GBM have been described.

Recent studies suggest that long noncoding RNAs (lncRNAs) can be utilized as peripheral biomarkers in several cancers due to their stable secondary structures. LncRNAs are 200 nucleotide or greater in length and do not encode for any protein [5]. LncRNAs act as interface between DNA and specific chromatin remodeling activities. They are regulators of cancer initiation and progression and may outnumber protein-coding genes, representing a largely unexplored functional component of the genome. LncRNAs transcribed from intergenic regions, translocate to different genomic loci (*trans* action) and regulate the expression of oncogenes and tumor suppressor genes in tissue- and cell-specific manners [6].

One important lncRNA is HOX Transcript Antisense Intergenic RNA, or HOTAIR, a well-characterized long noncoding RNA belonging to the homeobox superfamily [7]. HOTAIR is transcribed from the HOXC locus on chromosome 12q13.13 [7]. Numerous studies have shown that HOTAIR plays a critical role in multiple cancers, including melanoma, breast cancer, esophageal squamous cell carcinoma, bladder cancer, and pancreatic cancer [7–10].

We have previously demonstrated that HOTAIR is overexpressed in GBM and it controls GBM cell proliferation [5]. Knockdown of HOTAIR reduced proliferation and increased apoptosis of GBM cells in vitro and in vivo [5]. Importantly we demonstrated that HOTAIR is part of the proliferative pathway controlled by bromodomain reader proteins, which are therapeutic targets in GBM and other cancers [5]. Bromodomain Containing 4 (BRD4) protein controlled HOTAIR levels by binding to the HOTAIR promoter. Inhibition of BRD4 activity with small molecule Bromodomain and Extraterminal (BET) inhibitors reduced BRD4 binding at the HOTAIR promoter and reduced HOTAIR levels in vitro and in vivo [5]. In addition, overexpression of HOTAIR was able to rescue the anti-proliferative effects of BET inhibitors on GBM cell proliferation. Collectively, these studies suggested that HOTAIR can be used as a biomarker for responsiveness of GBM cells to BET inhibitors and potentially other targeted therapies.

As we found that HOTAIR is a biomarker of GBM cells to treatment response of BET inhibitors and that HOTAIR expression is completely undetectable in healthy patients brain we hypothesized that HOTAIR levels may be used as a peripheral biomarker in GBM patients. Furthermore, HOTAIR has been shown to be a possible serum biomarker in several cancers [8, 9, 11, 12]. In this study we demonstrate that HOTAIR levels is a serum biomarker in GBM. HOTAIR levels are higher in serum from GBM patients relative to the corresponding controls. Therefore, serum HOTAIR levels can be potentially be monitored in GBM patients during treatment to detect treatment response and tumor recurrence.

## Results and discussion

### HOTAIR expression is higher in serum isolated from GBM patients relative to controls

**Fig. 1:**
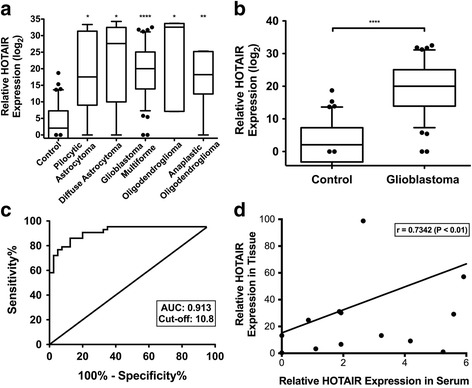
HOTAIR is detected in serum of GBM patients. **a** HOTAIR levels are higher in serum from brain tumor patients relative to controls. Serum from normal patients (*n* = 40) or patients suffering from either pilocytic astrocytoma (*n* = 6), diffuse astrocytoma (n = 6), GBM (*n* = 43), oligodendroglioma (n = 4), anaplastic oligodendroglioma (*n* = 7), were analyzed for the levels of HOTAIR. HOTAIR levels were detected by qRT-PCR and normalized to the corresponding GAPDH levels and relative to an internal control serum. **b** HOTAIR expression in GBM serum is significantly higher than in normal control patients. The expression level of serum HOTAIR in 43 GBM and 40 control patients were detected and analyzed using Mann-Whitney test (*P* < 0.001). **c** Serum HOTAIR can be a significant diagnostic indicator for GBM patients. Serum HOTAIR can discriminate GBM and normal patients with an area under the ROC curve value of 0.913 (*P* < 0.0001). Note that sensitivity and specificity of the assay was 86.1% and 87.5%, respectively. **d** Serum HOTAIR levels correlate with GBM tumors. The HOTAIR levels in 15 paired tumors and serum from GBM patients were measured by qRT-PCR. *Pearson* correlation analysis demonstrated a medium correlation of serum HOTAIR levels and the corresponding tumor HOTAIR levels (*r* = 0.7342, *P* < 0.01)

HOTAIR expression has been shown to be greater in tumors from GBM patients relative to lower grade glioma. Therefore, we hypothesized that levels of HOTAIR would be higher in the serum from GBM patients relative to those from low grade brain tumors. To test this, we performed qRT-PCR to evaluate HOTAIR expression in serum from different brain tumors patients, including 6 pilocytic astrocytoma, 6 diffuse astrocytoma, 4 oligodendroglioma, 7 anaplastic oligodendroglioma, and 43 GBM patients. As a control, HOTAIR expression in serum from 40 control patients who did not suffer from any brain tumor was also measured. Our findings suggest that serum HOTAIR levels was higher in serum from all brain tumor patients relative to control serum and HOTAIR expression in GBM serum is significantly higher than in normal control serum (Fig. 1a, b and Methods are explained in Additional file 1: Materials and Methods).

### Serum HOTAIR has good diagnostic value as a GBM biomarker

To assess the diagnostic ability of serum HOTAIR in distinguishing GBM patients from control patients we compared serum HOTAIR levels from 43 GBM patients and 40 controls. Our results indicate that HOTAIR is a promising biomarker in GBM with an AUC of 0.913 at the cut-off value of 10.8. The sensitivity and specificity of HOTAIR are 86.1% and 87.5% respectively (Fig. 1b, c). Therefore, serum HOTAIR can be utilized as a peripheral biomarker for GBM. The development of this minimally-invasive “liquid biopsy” technique could greatly benefit GBM patients.

### The levels of HOTAIR are positively correlated in tumors and serum isolated from GBM patients

**Fig. 2:**
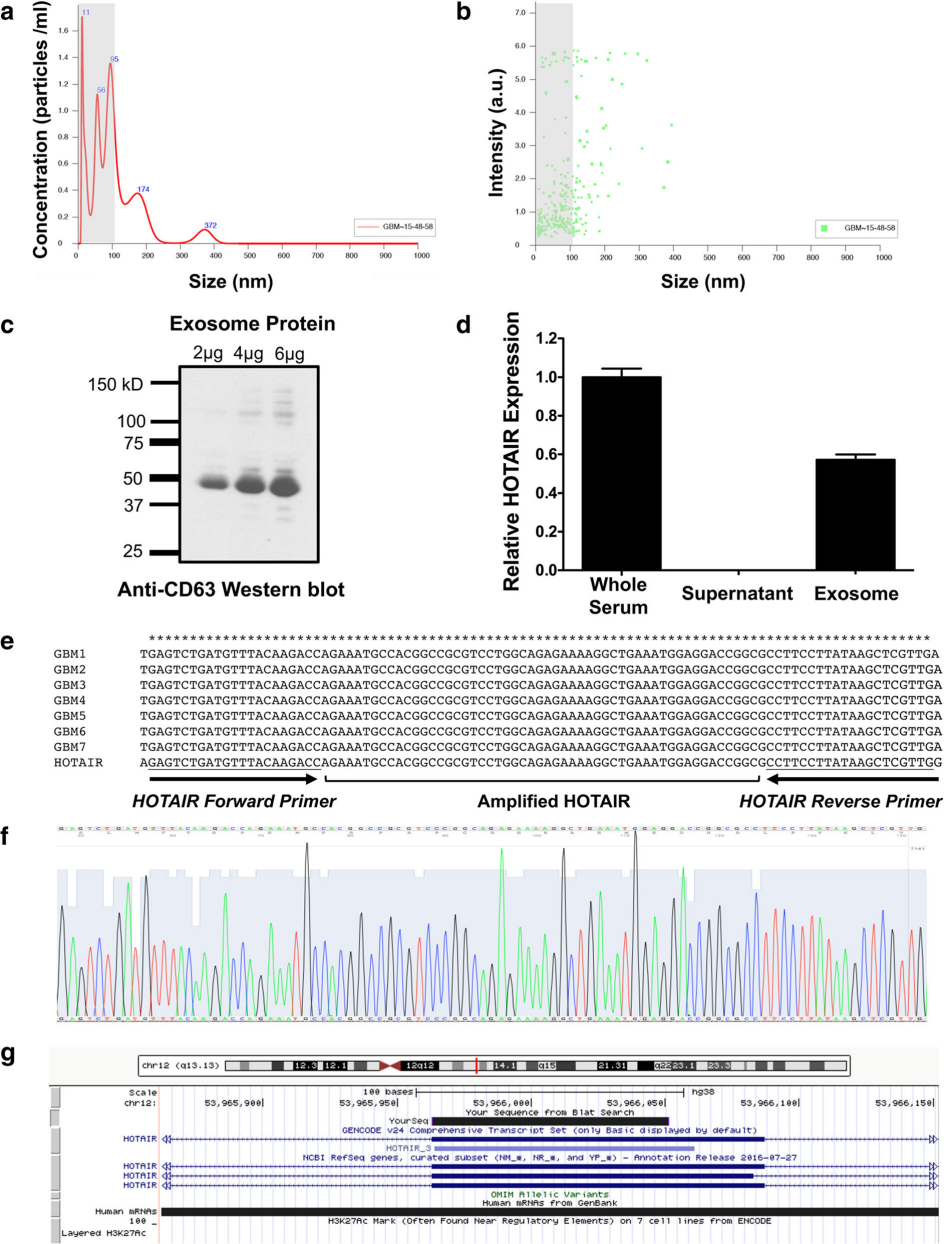
HOTAIR is enriched in exosome fraction of GBM serum. Exosomes were purified as previously described and the level of HOTAIR was measured by qRT-PCR. **a**, **b** Exosomes were found to be 100 nM or smaller (gray regions) in size as judged by NanoSight analysis. **c** Exosome-enriched protein CD63 was detected by Western blot analysis. **d** HOTAIR was present in whole serum and purified exosomes but not in serum supernatant depleted of exosomes. **e** Sequencing of HOTAIR qRT-PCR products amplified from GBM serum demonstrates the HOTAIR sequence. We amplified HOTAIR in GBM patient serum using qRT-PCR, followed by subcloning the corresponding PCR products into pCR™4-TOPO®TA vector and sequencing the inserts. We found that the inserts indeed contained HOTAIR sequence, **f** that it corresponds to the predicted HOTAIR sequence, and **g** mapped to the HOTAIR locus on the USC browser

To determine whether there is a correlation of HOTAIR levels in GBM tumors and serum we performed qRT-PCR for HOTAIR levels in 15 paired GBM tumor tissue and serum samples. As shown in Fig. 1d, we observed a moderate correlation of HOTAIR levels in tumors and serum (*r* = 0.7342, *P* < 0.01), suggesting that HOTAIR levels in serum may be derived from tumors. Consistent with this possibility we observed HOTAIR in exosomes purified from GBM patients (Fig. 2).

### HOTAIR is detected in exosomes present in GBM patient serum

Exosomes were purified from serum isolated from GBM patients and the level of HOTAIR was measured by qRT-PCR. Exosomes purified were found to be 100 nM or smaller in size (gray regions) as judged by nanoparticle tracking analysis (Fig. 2a, b). Exosome-enriched protein CD63 was detected by Western blot analysis, validating that exosomes were present in our samples. (Fig. 2c). HOTAIR was present in whole serum and purified exosomes but not in serum supernatant depleted of exosomes (Fig. 2d).

### Sequence of qRT-PCR products amplified from GBM serum maps to human HOTAIR locus

To validate our biomarker assay, we sought to determine whether the HOTAIR products we are amplifying contain the known HOTAIR sequence. To this end, we amplified HOTAIR from GBM patient serum using qRT-PCR, subcloned the corresponding qRT-PCR products into the pCR™4-TOPO®TA vector, and subsequently performed DNA sequencing of the cloned products. 7 *Taq* polymerase-amplified qRT-PCR products were picked randomly among all the samples and transformed into competent *E. coli*. Colonies were isolated the next day and plasmid DNA was isolated for subsequent sequencing. DNA sequencing demonstrated that the HOTAIR amplified from serum has the same sequence as the known sequence of HOTAIR. As shown in Fig. 2e, f and g, the sequence of all 7 HOTAIR inserts aligned to the HOTAIR locus exon at the USC browser. Furthermore, the amplified HOTAIR mapped to chromosome 12q13, which is the known location of HOTAIR in the human genome (Fig. 2g). As the HOTAIR gene has several splice variants, the PCR products amplified in this study detect HOTAIR splice variant HOTAIR-205. Collectively, these studies suggest that we are indeed measuring HOTAIR levels in our biomarker assay.

In this study we demonstrate that HOTAIR can be utilized as a peripheral biomarker for detecting GBM. HOTAIR, or HOX transcript antisense intergenic RNA, has been shown to be an important regulator of tumor growth and a possible serum biomarker in several cancers [5, 13]. First identified in 2007, HOTAIR is a polyadenylated RNA with 2158 nucleotides and 6 exons [7]. HOTAIR is transcribed from the Hox gene cluster [13]. The Hox genes, a subgroup of the homeobox superfamily, are 39 transcription factors classified in 4 clusters (HOXA, HOXB, HOXC, HOXD) that guide correct spatial organization during development. Aberrant expression of Hox genes has oncogenic effects, and many noncoding transcripts (miRNA and lncRNAs) have been detected in all Hox clusters [14]. HOTAIR is initially transcribed from HOXC locus, but later represses the expression of distal HOXD locus and other genes on chromosomes [7]. Importantly, HOTAIR has been found to *trans* act on multiple regions in the genome to regulate genes involved in metastasis and proliferation. Our own studies have demonstrated that HOTAIR controls GBM cell growth and we found that HOTAIR is highly expressed in GBM tumors yet completely undetectable in normal brain through single molecular sequencing (SMS) analysis [5]. This is in line with similar reports from other groups where HOTAIR was detected in the serum of patients suffering from melanoma, esophageal cancer, breast cancer, bladder cancer, and pancreatic cancer [7–11]. Hence, we propose that detection of the lncRNA termed HOTAIR in the serum of GBM patients can be utilized as a serum biomarker to detect GBM growth or recurrence.

Several proteins and microRNAs such as GFAP, lactate, miR-504, and miR-137 have been previously described as possible biomarkers for GBM [15–17]. However, a recent report by Vietheer et al. demonstrated that serum GFAP levels do not correlate with tumor recurrence in GBM in 33 samples in their studies [17]. Similar results were also observed for another protein, lactate, in serum [15]. Very few studies have been done on lncRNAs as biomarker in GBM. In the current study, serum lncRNA can be easily detected by qRT-PCR. Many groups are looking at lncRNAs now as they show highly promosing results in other cancers including melanoma, esophageal cancer, and breast cancer [7, 10]. Our study is one of the first studies to show that the levels of lncRNAs can be used as a biomarker in GBM. While most studies focus only on the use of biomarkers for predicting the survival of the GBM patients or prediagnostic alone, our studies focus on both diagnostic and prognostic value of HOTAIR.

Exosomes are small membrane-bound vesicles that are secreted into biological fluids such as blood, urine, saliva and milk from tumor cells. Recent studies have shown that RNAs, including lncRNA, microRNAs, and mRNAs are concentrated in these exosomes as they play a role in distance cell-to-cell communication. For instance, microRNAs in GBM cell derived exosomes have been postulated to control PTEN, SOX2, PI3K/AKT, STAT3 and ERK pathways [18]. We detected HOTAIR mainly in GBM serum exosomes, suggesting that HOTAIR could be possibly released from tumor tissue into serum in extracellular vesicles. Our initial studies suggest that freeze-thaw cycles affect the integrity of exosomes. Therefore, the levels of HOTAIR within exosomes were reduced after repeated freeze-thawing. However, the levels of HOTAIR within total serum were not affected. We postulate that the circulating HOTAIR we are detecting in serum is RNA and not DNA since amplifying HOTAIR from serum without reverse transcription yielded less PCR products relative to when reverse transcriptase was included (Additional file 2: Figure S1). In addition, we suggest that what we are amplifying from serum is indeed HOTAIR as DNA-sequencing of the qPCR products corresponded to the known HOTAIR sequence (Fig. 2e–g).

A longitudinal study of one recurrent GBM patient that was treated at our institution was performed to monitor HOTAIR expression in serum before and after tumor resection. We observed a reduction of serum HOTAIR levels after surgery and a further reduction at the 2 week post-surgery follow-up (Additional file 3: Figure S2). Although this is only one patient, this promising result suggests that further experiments are warranted to determine whether HOTAIR levels do indeed decrease after GBM tumor removal. Furthermore, prior studies have demonstrated that HOTAIR expression is greatest in GBM tumors with a mesenchymal classification [19]. However, we were unable to detect correlation of HOTAIR serum levels with any particular subgroup of GBM tumors (unpublished studies). Further studies are required to determine whether HOTAIR expression correlates with any particular tumor type in GBM. Similarly, more work is needed to determine whether HOTAIR serum levels can be used as a diagnostic peripheral marker for other brain tumor patients.

GBM, a WHO Grade IV tumor, is the most malignant primer tumor of the human central nervous system with an extremely poor prognosis. An efficient biomarker that can monitor GBM progression and treatment response will improve the clinical management of this lethal disease. Our studies suggest that we have developed a novel peripheral biomarker assay for GBM. To our knowledge this is the first report of a peripheral biomarker for GBM using a long noncoding RNA. This is also the first report that HOTAIR is a peripheral biomarker for GBM that is expressed in exosomes from serum of GBM patients. 

## Additional files

Additional file 1: Supplementary materials and methods. (DOCX 109 kb)

Additional file 2: **Figure S1.** HOTAIR expression detected in our biomarker assay is derived mostly from circulating RNA, not DNA. 3 GBM serum samples were selected at random and the relative HOTAIR expression in GBM serum with and without reverse transcription (RT)-PCR was determined. The HOTAIR RNA was reverse-transcribed into HOTAIR cDNA and qPCR was performed. The circulating HOTAIR DNA in the serum was detected by qPCR without RT. The considerable difference between HOTAIR expression with and without RT demonstrates that the HOTAIR we are detecting in our qRT-PCR reactions is derived from RNA and not DNA. (PDF 1003 kb)

Additional file 3: **Figure S2.** A longitudinal study on a single GBM patient was carried out in order to monitor the changes in serum HOTAIR expression over time. 3 different time points were included in this study: pre-op (the blood was drawn right before the surgery started), post-op (at least 24 h after surgery) and during the 2 week follow-up (F/U) with the neurosurgeon. We show that the level of HOTAIR decreases after surgery and at the follow-up visit. (PDF 430 kb)

## Abbreviations

AUC: Area under the curve
BET: Bromodomain and Extraterminal
BRD4: Bromodomain Containing 4
GBM: Glioblastoma multiforme
HOTAIR: HOX transcrpt antisense intergenic ribonucleic acid
LB: Luria broth
lncRNA: Long noncoding RNA
MRI: Magnetic resonance imaging
qRT-PCR: Quantitative real time-polymerase chain reaction
ROC: Receiver operating curve
SMS: Single molecular sequencing
