# Supplementary material for: Serum long noncoding RNA HOTAIR as a novel diagnostic and prognostic biomarker in glioblastoma multiforme
Source: Mol Cancer. 2018 Mar 20;17:74. doi: 10.1186/s12943-018-0822-0 (PMC5861620; doi:10.1186/s12943-018-0822-0)
Supplement: Supplementary file 1 — Supplementary materials and methods. (DOCX 109 kb) [file 12943_2018_822_MOESM1_ESM.docx]

**METHODS**

**Patient Selection and Sample Collection**

GBM tumor tissue, brain tumor patient serum and control patient serum were obtained from the Florida Center for Brain Tumor Research (FCBTR) and the University of Miami Brain Tumor Initiative (UMBTI). Blood samples were collected with full informed consent and agreement, approved by the Institutional Review Board of the University of Miami, and the University of Florida. Blood samples were collected from 43 GBM patients, 6 pilocytic astrocytoma patients, 6 diffuse astrocytoma patients, 4 oligodendroglioma patients, 7 anaplastic oligodendroglioma patients, and 40 controls (30 control serum samples were purchased from Bioserve Biotechnologies, Beltsville, MD and these patients did not have GBM as judged clinically). 15 paired serum and tumor tissues from GBM patients were obtained. None of the patients had received any clinical treatment before collection of the serum samples.

**Total RNA Isolation (Serum)**

Total RNA was extracted from the serum samples using TRIzol LS Reagent (Invitrogen Life Technologies) as previously described. Briefly, 400μl of patient serum was mixed with an equal volume of TRIzol LS Reagent and the mixture was incubated for 5 minutes on ice. Subsequently, 800μl of Acid-Phenol: Chloroform (Invitrogen Life Technologies) was added, vortexed for 30 seconds, and centrifuged for 25 min at 14000 rpm at 4°C to separate the mixture into aqueous and organic phases. The aqueous (upper) phase was carefully removed without disturbing the lower phase or the interphase and 1.25 volumes of room temperature 100% ethanol was added to the recovered aqueous phase. The serum RNA was then purified using mirVana PARIS RNA Purification kit (Invitrogen Life Technologies), washed once with 700uL miRNA Wash Solution 1 and twice with Wash Solution 2/3. The final RNA was recovered in 25μl of preheated (95°C) RNase-free water and stored at -80°C. The RNA concentration and quality was assessed using the NanoDrop ND-1000 Spectrophotometer (Thermo Fisher Scientific).

**Total RNA Isolation (Tissue)**

Tumor tissue was incubated with TRIzol Reagent (Invitrogen Life Technologies) at room temperature for 5 minutes and mixed with 400ul of chloroform. The mixture was shaken vigorously by hand for 15 seconds and incubated at room temperature for 2 minutes. Subsequently, the mixture was centrifuged at 12000x*g* for 15 minutes at 4°C. 500μl of the top aqueous layer was recovered and added to an equal volume of 70% ethanol, vortexed vigorously, and then purified by QIAGEN RNAeasy Kit (Thermo Fisher Scientific) following the manufacturer’s instructions. 280μl of DNase solution (Qiagen) was added for digestion of DNA. The final RNA was eluted in 50ul RNase-free water and placed on ice immediately.

**Reverse Transcription**

Complementary RNA (cDNA) was synthesized from total RNA recovered using the High Capacity cRNA Reverse Transcription Kit (Applied Biosystems) following the manufacturer’s instructions. Briefly, a total of 37ng RNA was utilized as template in a total volume of 20.0μl consisting of 0.8μl 100mM dNTP mix, 2.0μl 10X RT Buffer, 2.0μl 10X RT Random Primers and 1.0μl MultiScribe Reverse Transcriptase. The conditions for RT reactions are 25°C for 10 minutes, 37°C for 2 hours and lastly 85°C for 10 minutes.

**Quantitative Real Time-Polymerase Chain Reaction (qRT-PCR)**

To detect HOTAIR in serum, we performed sequential qRT-PCR reactions. Each reaction contained 4.0μl cDNA, 5.0μl *Power* SYBR® Green PCR Master Mix (Applied Biosystems), 0.5μl SYBR Green forward primer and 0.5μl SYBR Green reverse primer. The cDNAs were amplified using QuantStudio^TM^ 6 Flex Real-Time PCR System thermal cycler (Thermo Fisher Scientific). The pre-designed qRT-PCR SYBR Green primers (Sigma-Aldrich) used were HOTAIR forward, 5’-GAGTCTGATGTTTACAAGACC-3’; HOTAIR reverse, 5'-CAACGAGCTTATAAGGAAGG-3’; The housekeeping gene used in the qRT-PCR reaction was GAPDH and the sequences are: GAPDH forward, 5'-ACAGTTGCCATGTAGAGG-3’; and GAPDH reverse, 5'-TTTTTGGTTGAGCACAGG-3’. The HOTAIR expression was normalized to corresponding GAPDH and to an internal control C1. The relative HOTAIR expression was calculated using 2^- ∆∆Ct^ method, where ∆Ct = Ct_target_ – Ct_reference_.

**DNA Cloning and Sequencing**

7 fresh qRT-PCR products were randomly isolated and TOPO^®^ Cloning reaction was performed. 4μl of qRT-PCR sample was added to 1μl salt solution and pCR^TM^ 4-TOPO^®^ vector (Applied Biosystems), incubated for 5 minutes at room temperature, and the pCR^TM^ 4-TOPO^®^ construct was immediately transformed into One Shot^®^ TOP10 and DH5α^TM^-T1^R^ competent *E. coli*. The mixture was incubated on ice for 30 minutes, followed by heat-shock for 30 seconds at 42°C without shaking. We then added 250μl of room temperature S.O.C. medium and they were shaken horizontally (200 rpm) at 37°C for 1 hour. After that, the transformation was spread evenly on Luria Broth (LB) plates containing 50μg/mL ampicillin and incubated overnight shaking at 37°C. 2-4 colonies were isolated and cultured overnight in LB medium containing 50μg/mL ampicillin, followed by plasmid DNA isolation using the PureLink^®^ Quick Plasmid Miniprep Kit (Applied Biosystems) according to the manufacturer’s instructions. Lastly, the purified plasmid DNA was used for DNA sequencing using M13 Forward and M13 Reverse primers.

**Exosome Preparation**

Exosomes were harvested from 1mL of GBM patient serum using 0.2 volumes Total Exosome Isolation (from serum) reagent (Invitrogen Life Technologies). The mixture was mixed well by vortexing until a homogenous solution was attained. Subsequently, the solution was incubated at 4°C for 30 minutes. Next, the sample was centrifuged at 10000x*g* for 10 minutes at 4°C. The supernatant was then aspirated and the pellet was resuspended in 400μl of PBS.

**Western Blot Analysis**

The exosome protein was quantitated by a Pierce^TM^ BCA Protein Assay Kit (Thermo Fisher Scientific) according to manufacturer’s recommendations. 2, 4 and 6μg of exosome protein were analyzed using 1-20% Tris-Glycine gels, and transferred to a nitrocellulose membrane. Membranes were probed with rabbit anti-CD63 IgG (1:1000, System Biosciences), and then incubated with anti-rabbit IgG-horseradish peroxidase (1:20000, System Biosciences) as secondary antibody. Bands were visualized on film by chemiluminescence using SuperSignal^TM^ West Dura Extended Duration Substrate (Thermo Fisher Scientific) per the manufacturer’s recommendations.

**Nanoparticle Tracking Analysis**

The isolated exosome protein was analyzed using the Nanosight NS300 system (Malvern Instruments Company). The exosome was diluted in sterile 1X PBS solution and the results were later analyzed using Nanosight NTA 2.3 Analytical Software.

**Statistical Analysis**

A Mann-Whitney test was performed to determine the significance of GBM serum HOTAIR levels relative to control serum. *Pearson* correlation analysis was performed to investigate the association between two continuous variables. In order to assess the diagnostic potential of serum HOTAIR, a receiver operating characteristics (ROC) curve was plotted and the area under the ROC curve (AUC), sensitivity, and specificity were evaluated. A P-value of <0.05 was considered statistically significant.
