# Supplementary figures and images for: Serum long noncoding RNA HOTAIR as a novel diagnostic and prognostic biomarker in glioblastoma multiforme
Source: Mol Cancer. 2018 Mar 20;17:74. doi: 10.1186/s12943-018-0822-0 (PMC5861620; doi:10.1186/s12943-018-0822-0)

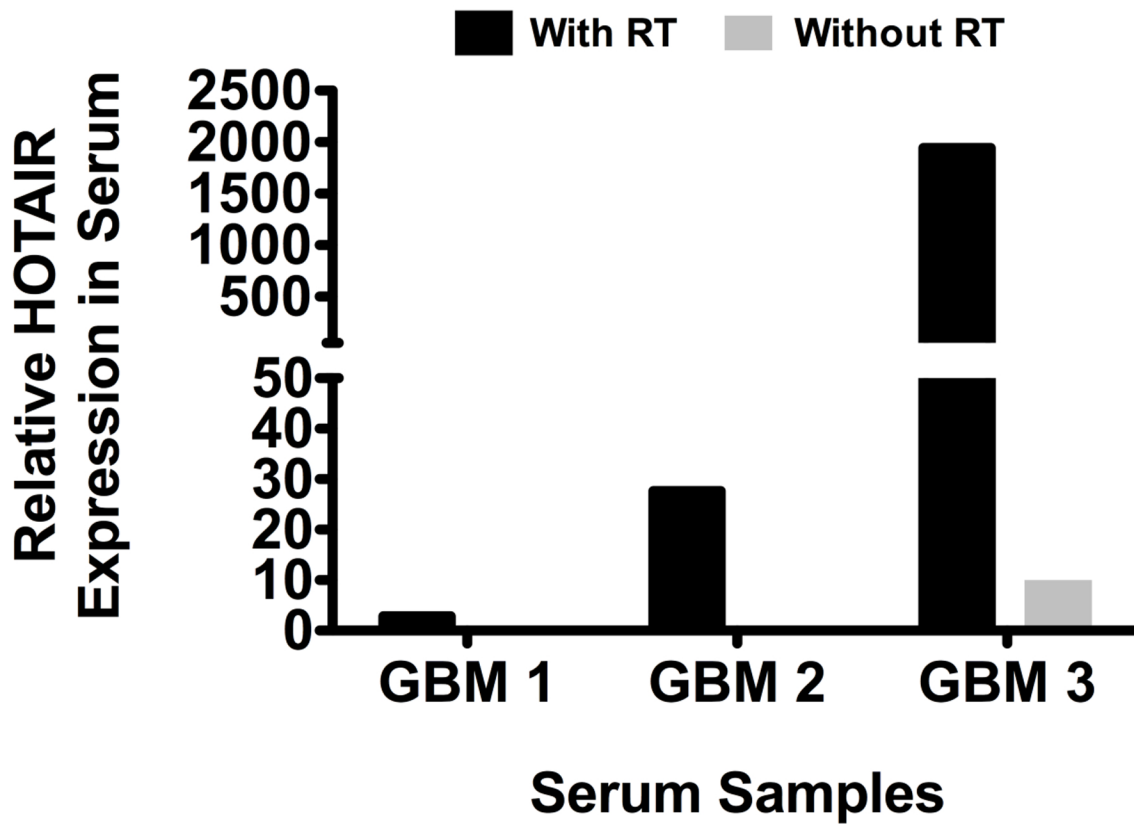

Supplement: Supplementary file 2 — Figure S1. HOTAIR expression detected in our biomarker assay is derived mostly from circulating RNA, not DNA. 3 GBM serum samples were selected at random and the relative HOTAIR expression in GBM serum with and without reverse transcription (RT)-PCR was determined. The HOTAIR RNA was reverse-transcribed into HOTAIR cDNA and qPCR was performed. The circulating HOTAIR DNA in the serum was detected by qPCR without RT. The considerable difference between HOTAIR expression with and without RT demonstrates that the HOTAIR we are detecting in our qRT-PCR reactions is derived from RNA and not DNA. (PDF 1003 kb) [file 12943_2018_822_MOESM2_ESM.pdf]

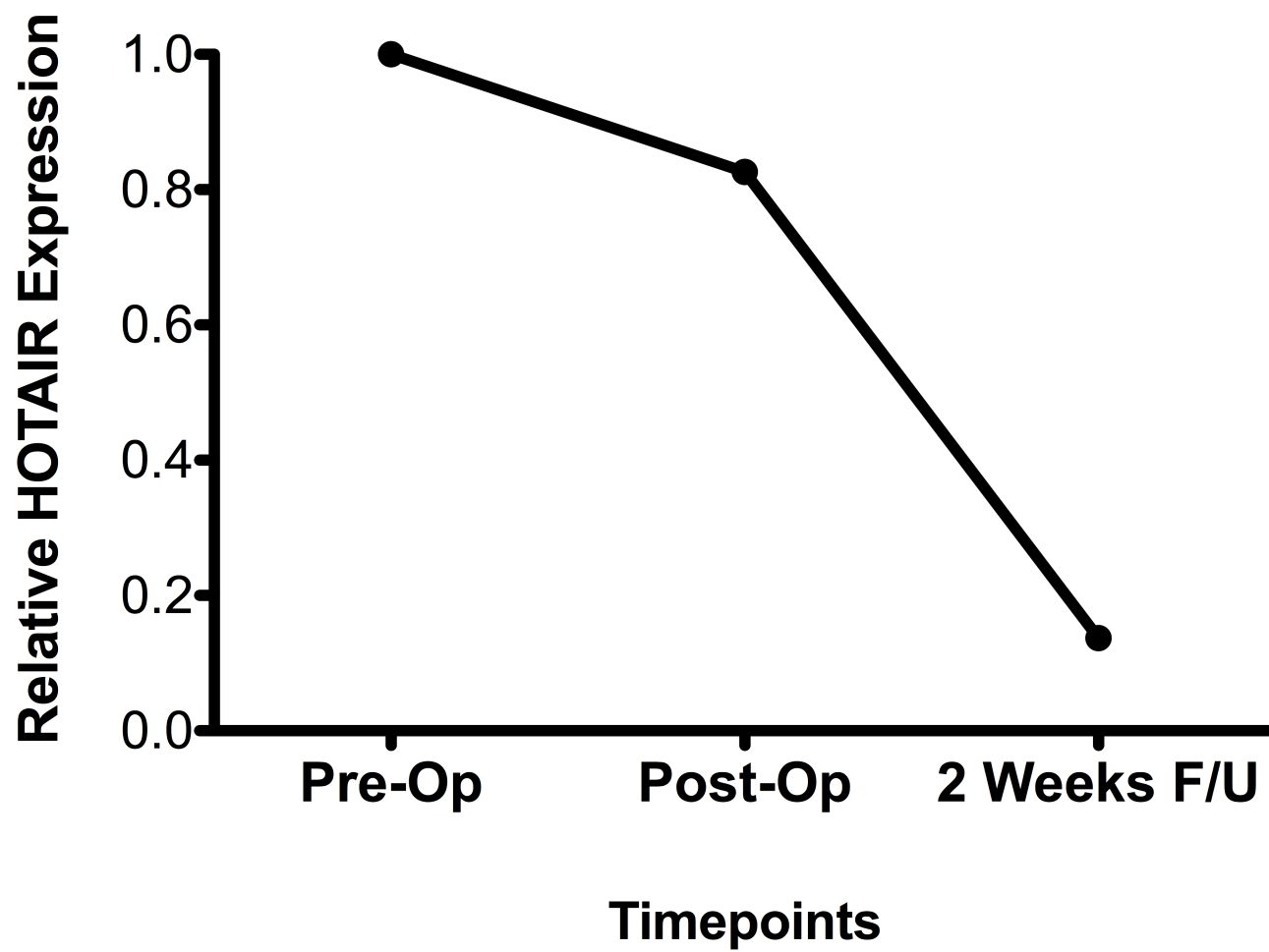

Supplement: Supplementary file 3 — Figure S2. A longitudinal study on a single GBM patient was carried out in order to monitor the changes in serum HOTAIR expression over time. 3 different time points were included in this study: pre-op (the blood was drawn right before the surgery started), post-op (at least 24 h after surgery) and during the 2 week follow-up (F/U) with the neurosurgeon. We show that the level of HOTAIR decreases after surgery and at the follow-up visit. (PDF 430 kb) [file 12943_2018_822_MOESM3_ESM.pdf]
